# Supplementary figures and images for: Direct Nanopore Sequencing of mRNA Reveals Landscape of Transcript Isoforms in Apicomplexan Parasites
Source: mSystems. 2021 Mar 9;6(2):e01081-20. doi: 10.1128/mSystems.01081-20 (PMC8561664; doi:10.1128/mSystems.01081-20)

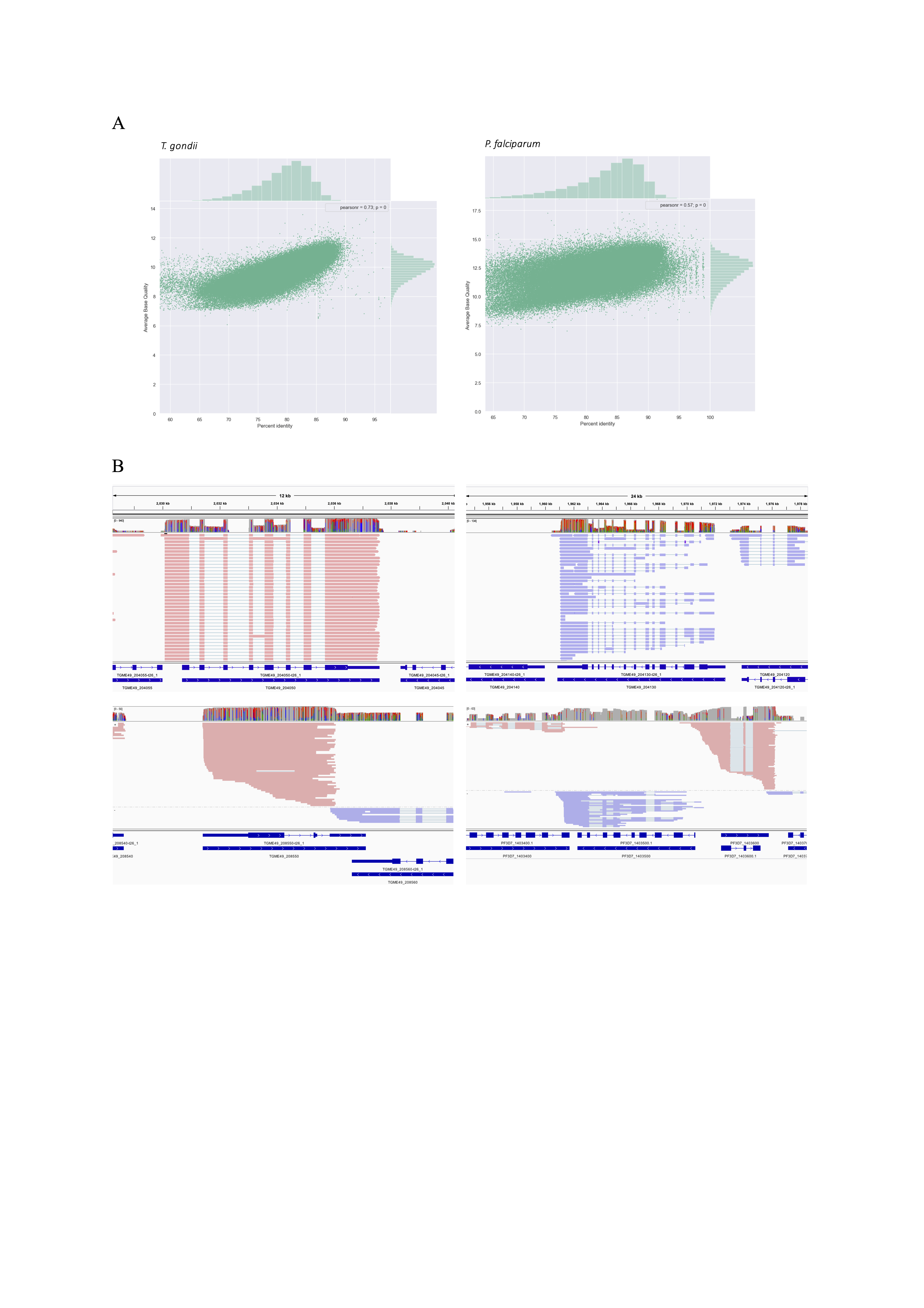

Supplement: FIG S1 [file msystems.01081-20-sf001.tif]

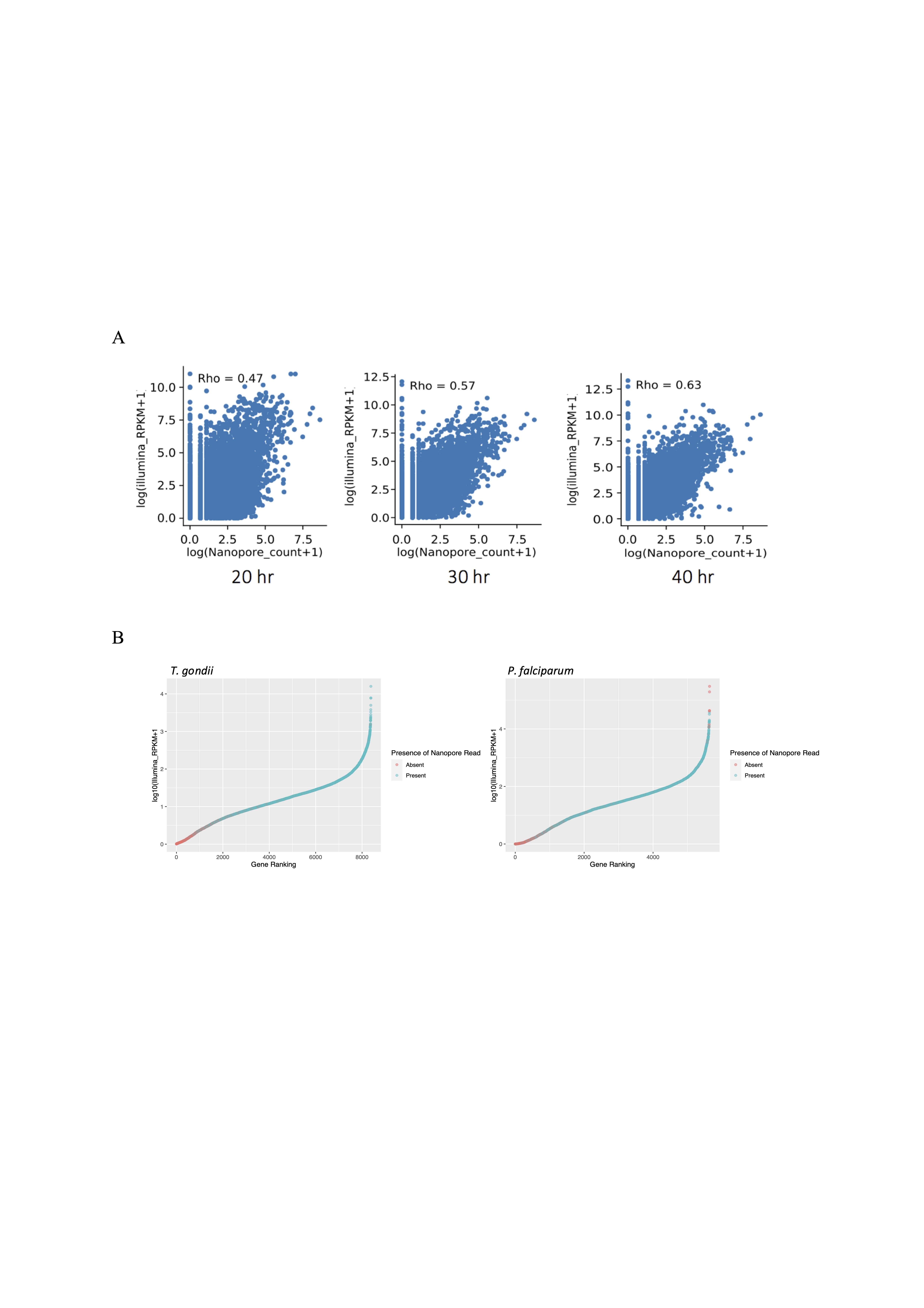

Supplement: FIG S2 [file msystems.01081-20-sf002.tif]

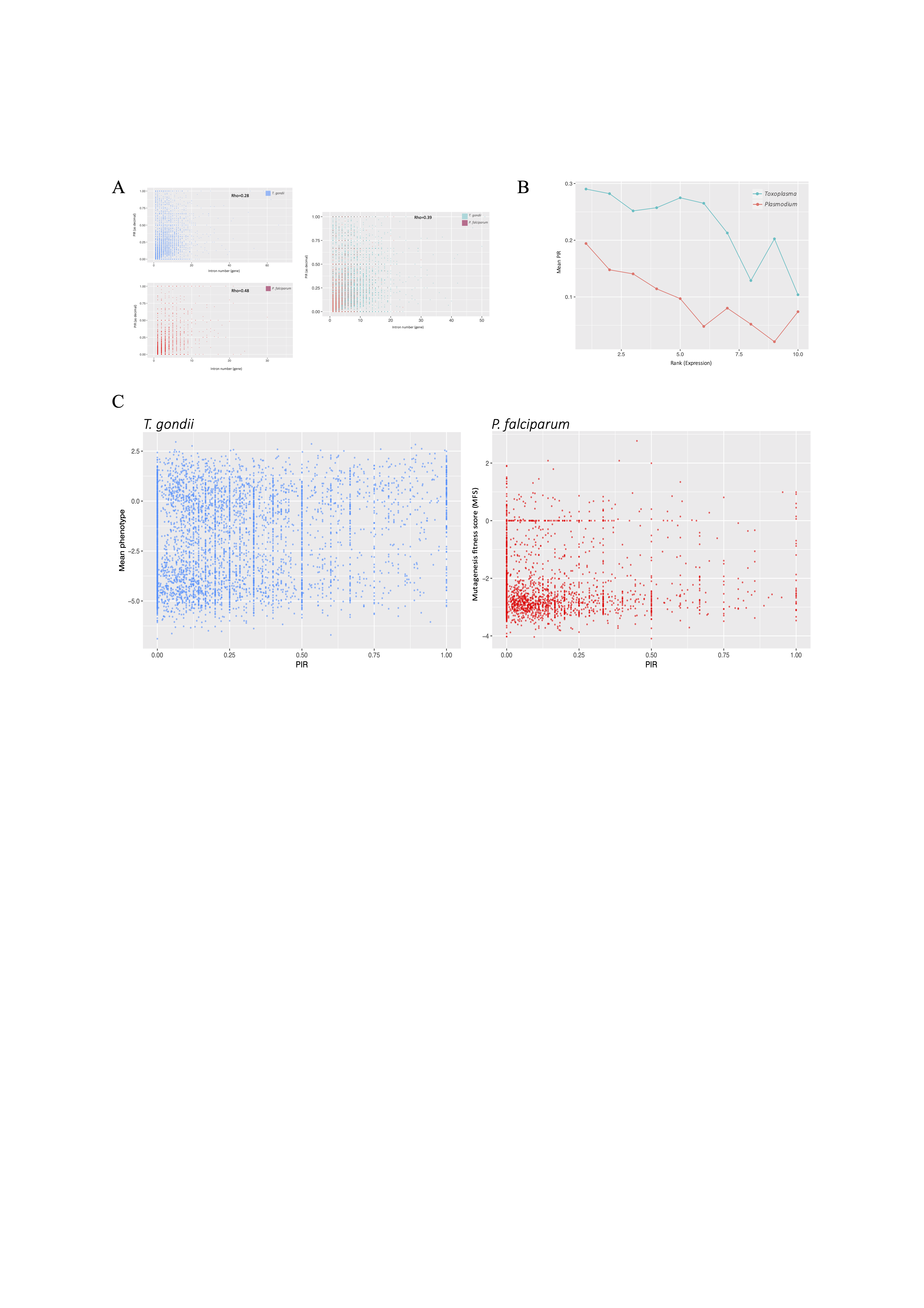

Supplement: FIG S3 [file msystems.01081-20-sf003.tif]

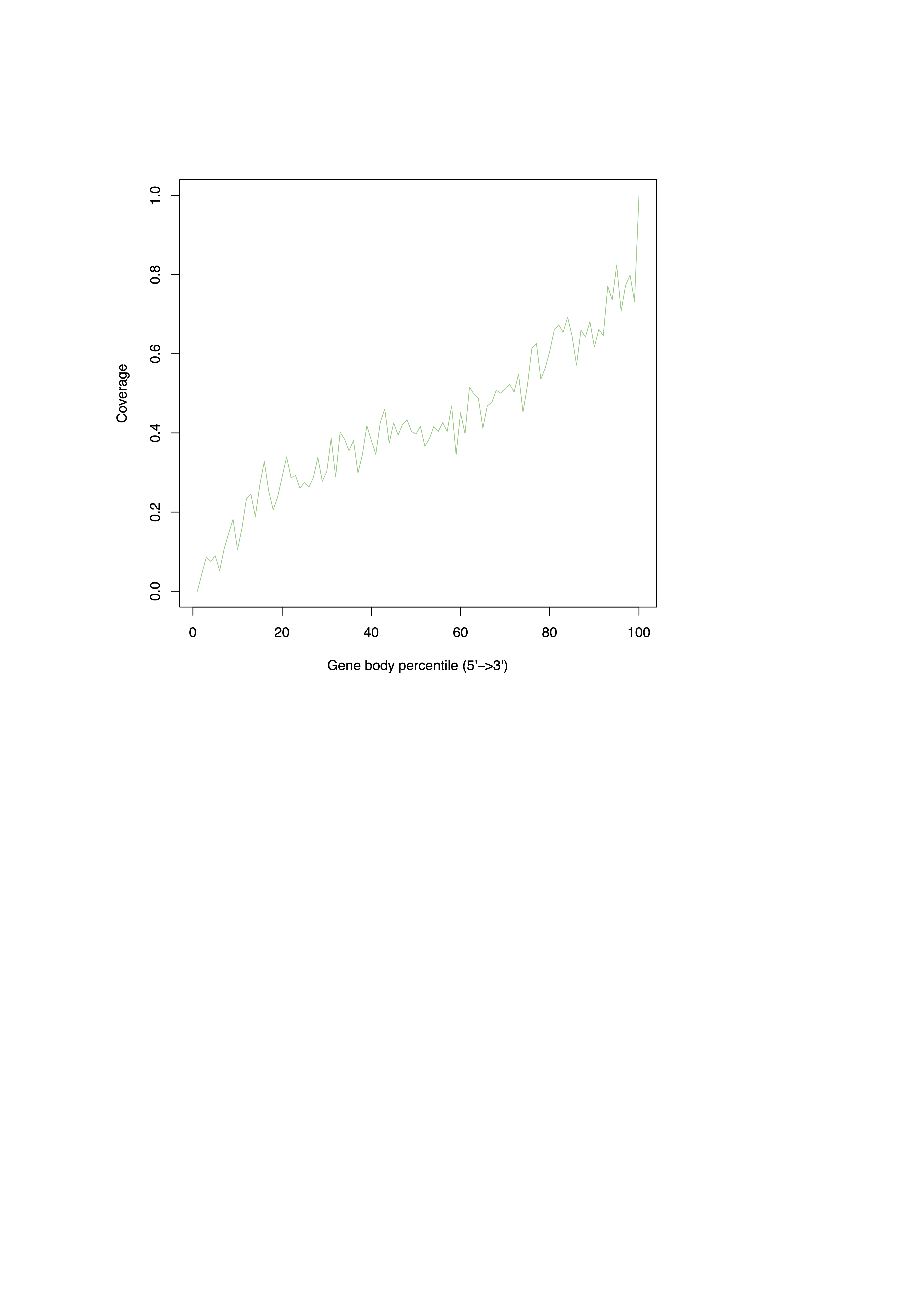

Supplement: FIG S4 [file msystems.01081-20-sf004.tif]

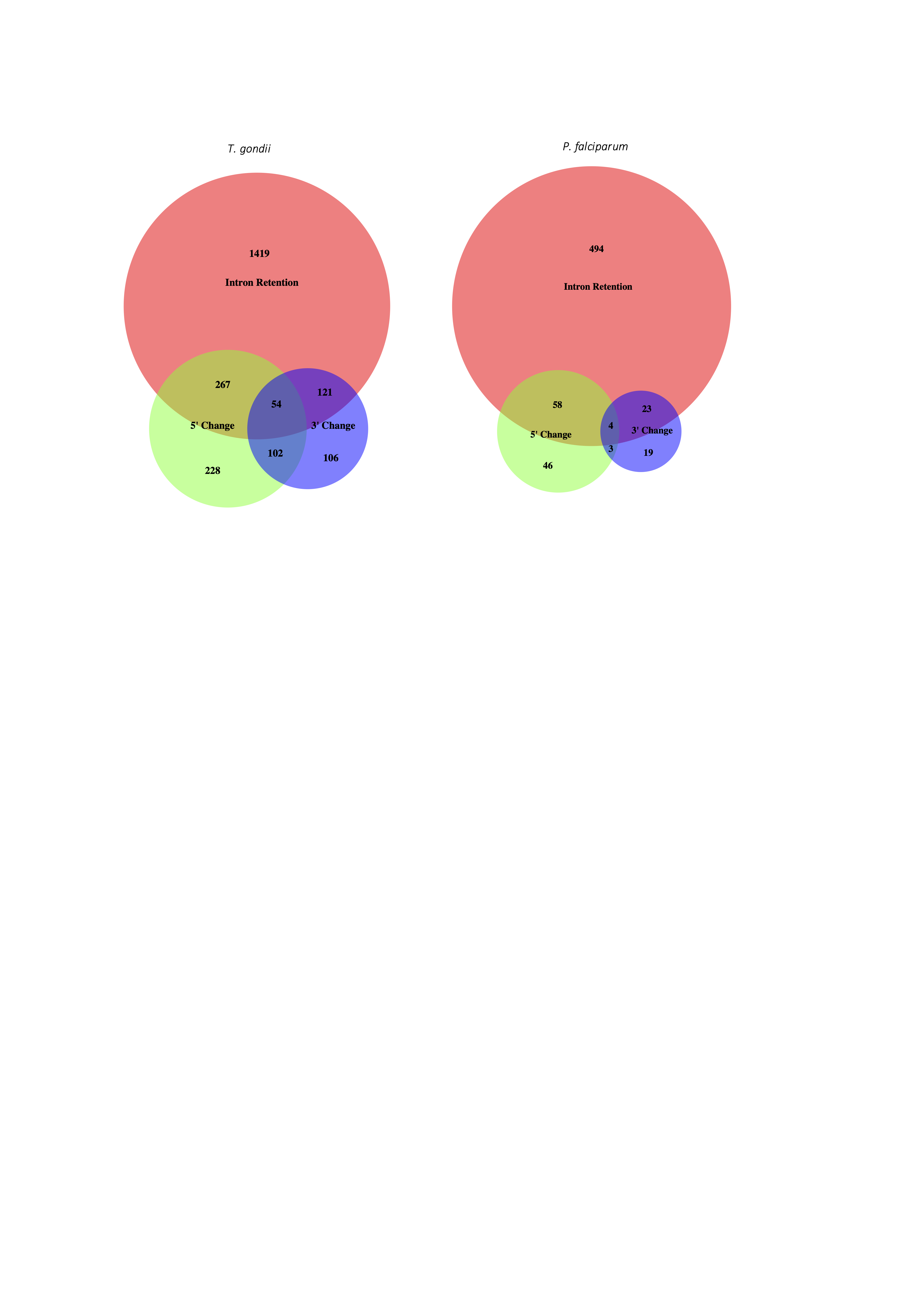

Supplement: FIG S5 [file msystems.01081-20-sf005.tif]
