# Supplementary material for: Direct Nanopore Sequencing of mRNA Reveals Landscape of Transcript Isoforms in Apicomplexan Parasites
Source: mSystems. 2021 Mar 9;6(2):e01081-20. doi: 10.1128/mSystems.01081-20 (PMC8561664; doi:10.1128/mSystems.01081-20)
Supplement: TEXT S1 [file msystems.01081-20-s0001.docx]

**Tool commands and description of analysis**

* Minimap2 was used for mapping ONT RNA reads to the genome and transcriptome of the parasites. The following commands were used.

*Toxoplasma*: ./minimap2 -ax splice -k14 -uf -G 5000 <ref.fa> <reads.fa> > <aln.sam>

*Plasmodium*: ./minimap2 -ax splice -k14 -uf -G 1500 <ref.fa> <reads.fa> > <aln.sam>

* HISAT2 was used for mapping Illumina RNA-seq reads to the genome and transcriptome of the parasites. The following commands were used.

*Toxoplasma*: hisat2 -x <ref-idx> -U <read.fastq> --max-intronlen 5000 -S <aln.sam>

*Plasmodium*: hisat2 -x <ref-idx> -U <read.fastq> --max-intronlen 1500 -S <aln.sam>

* Samtools was used for indexing and sorting mapped reads, as well as QC on mapping percentage. The following commands were used.

Indexing and sorting: ./samtools view -S -b sample.sam > sample.bam

./samtools sort sample.bam -o sample.sorted.bam

QC: ./samtools flagstat sample.sorted.bam

* Picard was used for merging mapped reads and QC on quality scores. The following commands were used.

Merging bam files: java -jar picard.jar MergeSamFiles \

I = <input_1.bam> \

I = <input_2.bam> \

O = <output_merged_files.bam>

QC: java -jar picard.jar QualityScoreDistribution \

I = <input.bam> \

O = <qual_score_dist.txt> \

CHART= <qual_score_dist.pdf>

* AlignQC was used for QC on error rate of mapped reads. The following command was used.

QC: alignqc analysis <reads.bam> -g <ref_genome.fa> -t <ref.gtf> -o

<output.xhtml>

* Wub was used for counting and correlation analyses of mapped reads. The following commands were used.

Read count (raw): bam_count_reads.py <sample.sorted.bam> -a 5 -z <ref.fasta> -t

<read_count.tsv>

Read count (RPKM): length_normalise_counts.py -f <ref> <input_counts>

<output_counts>

Correlate counts: correlate_counts.py -r <report.pdf> -L T <input_count_1>

<input_count_2>

* Bedtools was used for analysing genome coverage of mapped reads. The following command was used.

Genome coverage: bedtools genomecov -ibam <sample.bam> -bg -scale 1.0 >

<coverage.bedgraph>

* Deeptools was used for computing genome coverage ratios between different mapped read libraries. The following command was used.

Coverage ratio: bamCompare -b1 <sample1.bam> -b2 <control.bam> -of bedgraph -o

<output.bg> --binSize <> --scaleFactorsMethod readCount --ratio

log2 --pseudocount 1.0 --minMappingQuality 5

* FeatureCounts was used to count intron retained reads and junctions. The following command was used.

Intron count: featureCounts -t <gene/intron> -g <gene_id/intron_id> -a <ref.gff> -o

<output.txt> <input.bam> -L –minOverlap 6 -s 1

* Toolshed was used for extracting intron coordinates from annotation file. The following command was used.

Extracting intron coordinates: Gene BED To Exon/Intron/Codon BED expander

* RSeQC was used for counting junctions and analysing gene body coverage of mapped reads. The following commands were used.

Junction counts: junction_annotation.py –input-file <sample.bam> --refgene

<ref.bed> --min-intron 50 –mapq 5 -o <ouput>

Gene body coverage: geneBody_coverage.py -r <ref.bed> -i <sample.bam> -o

<output>

*FLAIR was used for analysing the productivity of intron retained and non-intron retained reads. The following commands were used.

Correct: python flair.py correct -q <mapped_reads.bed12> -g <ref_genome.fa>

Productivity: python predictProductivity.py -i <corrected_reads.psl> -g

<annotation.gtf> -f <ref_genome.fa> > <output.bed>

Intron presence: python mark_intron_retention.py <input.bed> <output.psl>

<out_coords.txt>

* Transcript coverage

Sequence and length of each mapped reads were extracted from bam file, matched to annotated length of genes based on gene id, and the coverage calculated. Fractions of full-length reads were calculated as the fraction of reads equalling or exceeding 95% coverage of a gene.

* Fold coverage

Fold coverage was calculated as the sum of read bases over the sum of all annotated gene bases.

*PIR/PSI calculations

PIR values were calculated as the proportion of alternative splicing events to the sum of reads for each junction/gene as identified by FeatureCounts. PSI values were calculated as the proportion of alternative splicing events to the sum of reads for each junction as identified by RSeQC. Types of alternative splicing were assigned based on whether either/both ends of the junction were within 5 nucleotide of the canonical splicing model, and the strandedness of transcript.
